# Association of Cardiac Autonomic Responses with Clinical Outcomes of Myasthenia Gravis: Short-Term Analysis of the Heart-Rate and Blood Pressure Variability

**DOI:** 10.3390/jcm11133697

**Published:** 2022-06-27

**Authors:** Monika Zawadka-Kunikowska, Łukasz Rzepiński, Małgorzata Tafil-Klawe, Jacek J. Klawe, Paweł Zalewski, Joanna Słomko

**Affiliations:** 1 Department of Human Physiology, Nicolaus Copernicus University Ludwik Rydygier Collegium Medicum in Bydgoszcz, Karłowicza 24, 85-092 Bydgoszcz, Poland; malg@cm.umk.pl; 2 Sanitas-Neurology Outpatient Clinic, Dworcowa 110, 85-010 Bydgoszcz, Poland; luk.rzepinski@gmail.com; 3 Department of Neurology, 10th Military Research Hospital and Polyclinic, Powstańców Warszawy 5, 85-681 Bydgoszcz, Poland; 4 Department of Hygiene, Epidemiology, Ergonomy and Postgraduate Education, Ludwik Rydygier Collegium Medicum in Bydgoszcz Nicolaus Copernicus University in Torun, M. Sklodowskiej-Curie 9, 85-094 Bydgoszcz, Poland; jklawe@cm.umk.pl; 5 Department of Exercise Physiology and Functional Anatomy, Ludwik Rydygier Collegium Medicum in Bydgoszcz Nicolaus Copernicus University in Torun, M. Sklodowskiej-Curie 9, 85-094 Bydgoszcz, Poland; p.zalewski@cm.umk.pl (P.Z.); jslomko@cm.umk.pl (J.S.); 6 Centre for Preclinical Research, Department of Experimental and Clinical Physiology, Warsaw Medical University, 1b Banacha Street, 02-097 Warsaw, Poland

**Keywords:** myasthenia gravis, heart rate variability, blood pressure variability, sympathovagal ratio, cardiac autonomic dysfunction, baroreflex sensitivity

## Abstract

Introduction: The aim of the study was to assess cardiac and autonomic function in patients with myasthenia gravis (MG) and to explore its relationship with disease outcomes. Methods: Thirty-eight patients with an MG were enrolled (median age 40.5 years; median disease duration 5.5 years). Cardiovascular parameters, baroreflex sensitivity (BRS), spectral indices of short-term heart rate (HRV), and systolic blood pressure variability (SBPV) were compared with age- and gender-matched controls (*n* = 30). Cardiac autonomic function was assessed during the response to standing (tilt) and deep breathing tests (expiration/inspiration ratio-E/I). Results: HR and BP responses to the tilt test were similar in both groups. MG patients, as compared to controls, were characterized by altered SBPV at rest, significantly reduced HR response to the deep breathing test (*p* < 0.001), increased sympathovagal balance after tilt (delta LF/HF-RRI, *p* = 0.037), and lower values of BRS (*p* = 0.007) and hemodynamic parameters, i.e., cardiac index, index contractility, left ventricular work index, at rest and during tilt. There was no association between disease duration and autonomic parameters. Disease severity, as determined by MGFA (Myasthenia Gravis Foundation of America) corrected for age and sex, was an independent predictor of diminished vagal tone (E/I ratio) and increased sympathetic response to tilt (delta LF/HF-RRI) as measured with HRV. Lower BRS was associated with greater disease severity and older age. Hemodynamic parameters were predominantly predicted by age and sex. Conclusion: Our results confirm cardiac autonomic dysfunction among MG patients with predominant parasympathetic impairment. Clinicians should consider evaluation of autonomic balance in MG patients with, or at risk for, cardiovascular disease.

## 1. Introduction

Myasthenia gravis (MG) is a rare, autoimmune, and T helper cells-mediated neurological disease characterized by fluctuating fatigability and weakness of extraocular, bulbar, respiratory and limb muscles [1]. Clinical presentation of MG is highly heterogeneous, depending on the patient’s age at onset, the autoantibody status, disease severity, thymic pathology, and underlying comorbidities and treatment [2,3]. Acetylcholine receptor (AChR) antibodies, muscle-specific kinase (MuSK) antibodies, and lipoprotein receptor-related protein 4 (LRP4) antibodies play an important role as mediators of neuromuscular junction damage in the pathogenesis of MG [4]. Previous studies suggest an autoimmune inflammation target for both skeletal and cardiac muscles in MG [5,6].

Localized or general muscle weakness are the cardinal symptoms of MG, however, some patients may experience various non-motor symptoms such as sleep abnormalities [7], cognitive deficits [8], and autonomic dysfunction (AD) [9,10]. Most of them are characterized as a consequence of cholinergic deficits. Both the chronic inflammatory process and the presence of cross-reactivity between muscle and ganglionic AChR antibodies have been considered crucially involved in the etiology of autonomic neuropathy [11,12]. However, it still remains unknown whether the presence of subclinical ANS dysfunction is, to some extent, due to parallel progression of muscle weakness and/or a consequence of MG treatment.

The most life-threatening ADs are related to the cardiovascular system and include decreased heart rate variability (HRV), cardiac arrhythmias, and even atrial fibrillation [6,12,13]. Considering that MG patients have altered baroreflex sensitivity (BRS) [14,15,16], it makes them more vulnerable to blood pressure fluctuations. Lower blood pressure variability (BPV) is associated with higher risks of cardiovascular events and morbidity [17,18]. Despite cardiac autonomic dysfunction (CAD) not routinely being diagnosed in MG patients, it is increasingly recognized as an important determinant of subclinical cardiac involvement [9]. It was described in different groups of patients with MG, with [14] and without thymoma [15,16,19], as well as/and in those in myasthenic crisis [20]. Previous evidence using electrophysiological and functional imaging methods showed both sympathetic and parasympathetic nervous system abnormalities and subclinical alterations in cardiac left ventricular function in MG [21,22,23,24,25].

Few studies in different MG subgroups using cardiovascular reflex tests, as well as HRV measures, have revealed mainly parasympathetic than sympathetic cardiac impairment [14,26], whereas others demonstrated sympathetic hyperactivity [15,19,26]. Thus, AD may considerably affect cardiovascular function, which increases cardiovascular risk and poor prognosis. Interestingly, fatigue and poor exercise capacity in MG may overlap the symptoms of CAD and lead to delayed recognition of cardiac complications, as well as rehabilitation management [6].

Impedance cardiography (ICG) is a simple, non-invasive method of hemodynamic parameters, left ventricular function, myocardial contractility, and thoracic fluid content. Most studies have demonstrated a good correlation between ICG, echocardiography, and invasive diagnostic techniques [27]. Only a few studies so far have assessed cardiac autonomic and hemodynamic functions in mild and moderate severity MG patients. To our knowledge, there is no data exploring comprehensively and simultaneously, the short-term analysis of the HRV, BPV, BRS, and clinical outcomes of MG patients.

The primary aim of this study was to compare cardiac and autonomic function between patients with MG and healthy controls (HCs). An additional purpose was to assess whether cardiac and autonomic measures are associated with clinical outcomes in individuals with MG.

## 2. Material and Methods

### 2.1. Study Participants and Study Protocol

It was a cross-sectional and case-control study conducted between January 2017 and March 2022. All subjects gave their written informed consent for this study, which was approved by the Bioethical Committee of Collegium Medicum in Bydgoszcz, Nicolaus Copernicus University in Torun (KB 747/2017). The study included 38 patients with MG aged 19–69 years (5 males, 33 females) and 30 age-matched healthy control subjects (HCs) (7 males, 23 females). All patients underwent a detailed clinical and neurological examination in the outpatient clinic (Sanitas, Bydgoszcz) by the same neurologist (Ł.R.). All HCs were normal in the neurological examination. Inclusion criteria for the MG patients were: age above 18 years old, diagnosis of MG, and no disease exacerbation within 90 days preceding the study. The inclusion criteria for controls included the following: no history of autoimmune disorders or neurological diseases. The exclusion criteria for MG patients and controls included: past history of cardiac disease; arrhythmia, including atrial fibrillation; the presence of major concurrent illness (respiratory involvement or in state of MG crisis); diabetes, hypertension, hyperthyroidism, hypothyroidism, or any other disease that might affect the autonomic nervous system; treatment with beta-blockers, antihypertensive drugs. In all patients, the cardiac autonomic function was assessed while receiving treatment for MG. Demographic and clinical data were obtained from medical records (Table 1). HCs were recruited from the local community (northern Poland).

The diagnosis of MG was established by characteristic clinical presentation (fluctuating weakness of ocular and/or extraocular muscles) and at least one of the following criteria: positive AChR autoantibodies (AChR-Abs), or MuSK antibodies (Musk-Abs), electrophysiological findings (repetitive stimulation and/or single-fiber electromyography), and clinical response to cholinesterase inhibitors. All of the patients had a stable course of disease for a minimum three months period.

Patients were categorized into subgroups according to age at onset (early-onset MG < 50 years of age, late-onset MG > 50 years of age), the clinical disease type (ocular MG symptoms restricted to the ocular muscles or generalized MG confirmed by the involvement of extraocular muscles), antibody status (AChR-Abs, Musk-Abs, seronegative), current MG therapy, thymic pathology and history of thymectomy [4]. Serum levels of AChR-Abs were detected by enzyme-linked immunosorbent assay (ELISA). IgG4 antibodies against MuSK were measured by ELISA in subjects lacking anti-AChR antibodies. Myasthenic exacerbation was defined as the clinical deterioration of previously reported muscle weakness lasting more than 24 h unrelated to fever and/or infection, resulting in an increase in the MGFA classification by at least one class. The worsening of symptoms within the last 30 days were considered as a single exacerbation [16]. Thymic pathology was assessed in accordance with the chest CT scan results and available histology findings.

The Myasthenia Gravis Foundation of America (MGFA) classification was developed as a tool for assessing disease severity. The MGFA classes are pure ocular weakness (class I), mild-generalized weakness (class II), moderate generalized weakness (class III), severe generalized weakness (class IV), and intubation/myasthenic crisis (class V). Within generalized MG (II-IV), patients are classified as class A (predominant limb/axial muscles involvement) or class B (predominant bulbar-oropharyngeal/respiratory muscles involvement) [28,29].

### 2.2. Cardiovascular Autonomic Function Test

The cardiac and autonomic functioning of participants was recorded using the TFM software version 2.3.20.20 (TFM, CNSystems Medizintechnik, Graz, Austria). In the present study, recordings were performed in a supine position for a minimum of 10 min and during the head-up tilt test (HUTT), using a 70° angle of tilt for 5 min. All the functions of TFM have been validated and successfully used in recent clinical studies [30,31,32].

TFM integrates ECG signal (electrocardiography), oscillometric, continuous plethysmographic blood pressure registration, and ICG (impedance cardiography), allowing analysis of the power spectral analysis of HRV and SBPV. Heart rate (HR) was obtained from the ECG. Continuous arterial systolic (sBP), diastolic (dBP), and mean (mBP) blood pressure were measured noninvasively beat-to-beat by finger plethysmography on the right hand. The finger blood pressure values were automatically calibrated (every 3 min) against oscillometric upper left arm measurements of arterial blood pressure [30]. In general, four electrodes are used for the impedance measurement: one placed on the neck, two electrodes lateral on the thorax, one neutral electrode on the right arm. ICG provides measures of cardiac function (cardiac index, CI = CO/body surface; stroke volume index, SI = SV/body surface), in addition to indices of LV myocardial function/contractility [index of contractility, IC = ΔZ/Δt_max_; left ventricular work index, LVWI = constant × (mean blood pressure − pulmonary artery occlusion pressure) × CI]. Thoracic fluid content (TFC = 1/kOhm]) was measured as an indicator of the volume of thoracic intravascular and extravascular fluid in the chest cavity. The afterload was calculated as the total peripheral resistance index (TPRI = mean BP/cardiac index) and preload as the end-diastolic index (EDI).

Sympathetic reactivity was assessed with the HUTT, while parasympathetic reactivity was evaluated by testing HR response to deep breathing (DBT).

During DBT, subjects were instructed to breathe slowly at 6 breaths/min (5 s inspiration and 5 s expiration). The E/I-ratio was calculated as the mean value of the longest R-R interval during expiration divided by the mean value of the shortest R-R interval during inspiration, averaged over 6 cycles. E/I was abnormal if it was ≤1.11 [33].

The ANS function was evaluated by baroreceptor sensitivity (BRS) using the sequence method and the power spectral analysis of the short-term heart rate (HRV) and systolic blood pressure variability (SBPV) applying an autoregressive (AAR) methodology as described by Bianchi et al. [31].

TFM automatically calculates a set of variables accounting for the variability of the heart rate (RR interval) and blood pressure: low frequency (LF, 0.04–0.15 Hz), high frequency (HF, 0.15–0.40 Hz), total power spectral density (PSD), representing total variability. For analysis, results were expressed in absolute and normalized values (PSD-RRI, LFnu-RRI, HFnu-RRI, LF-RRI, HF-RRI for HRV and PSD-sBP, LFnu-sBP, HFnu-sBP, LF-sBP, HF-sBP for SBPV). To assess sympathovagal balance, the ratios between LF and HF bands for heart rate and blood pressure variability was also calculated (LF/HF, LF/HF-RRI, LF/HF-sBP) [34].

As previously described, the LF band reflects contributions from both the parasympathetic and sympathetic modulation of the sinoatrial node (LF-RRI) and sympathetic modulation of the vascular tone (LF SBPV), while the HF band reflects parasympathetic modulation of cardiovascular activity influenced by respiration [34].

Baroreceptor sensitivity (BRS) was calculated using the sequence method as the slope of the linear regression between beat-to-beat sBP values (mmHg) during 10 min of supine rest [32].

All measurements were performed at the same time of the day, between 8–12 a.m., in a quiet, darkened room with a standard temperature (22 ± 1 °C) and air humidity, by the same investigator [M.Z.K.]. All participants were instructed to refrain from alcohol and caffeine consumption, smoking, and intensive exercise for at least 12 h before testing [35].

### 2.3. Statistical Analysis

In our study, HRV and BPV data were exported from the TFM software into Microsoft Excel for further analysis and then transferred Statistica. (version 13.3, StatSoft, Kraków, Poland) The AAR model may produce outliers when analyzing RR intervals, thus, all HR beat-to-beat data were filtered using Grubbs’s test for outliers’ elimination. This method of filtering is well-described and has a strong mathematical background [36].

All data are presented as median (lower quartile–upper quartile). The data distribution of the study variables was verified with the Shapiro–Wilk test. Differences in the distribution of qualitative variables were determined with the Chi-square test, while the differences in quantitative variables were determined with the use of the nonparametric Mann–Whitney test. The strength and significance of the correlation between selected variables were calculated using the nonparametric Spearman’s test. The multiple regression model, based on three predictors (age, sex, MGFA class), was also used in order to determine significant predictors for cardiac, BRS, HRV, and SBPV variables. The level of significance for all tests was set at *p* < 0.05.

## 3. Results

The demographic and clinical characteristics of the study population are presented in Table 1. We included 38 patients with MG (median age of 40.5) and with female sex predominance (86.8%). The median duration of the disease was 5.5 years. There were no significant differences between the patient and the HCs with respect to age and sex distribution, *p* > 0.05. Early disease onset before the age of 50 occurred in 35 patients (92.1%), while 3 cases (7.9%) were late disease onset. With respect to AChR-Ab status, 23 (60.5%) patients were seropositive, whereas 15 (39.5%) were seronegative. MGFA class at the moment of testing was available for all patients. Eight (21.1%) patients presented ocular weakness (Class I); 19 (50.0%) mild symptoms (Class II); and 11 patients (28.9%) with moderate muscle weakness (Class III) was documented. Thymic pathology was detected in 22 (59.5%) MG patients and fourteen of them underwent thymectomy. Histopathological evaluation revealed thymoma in one case. Most patients classified and had a generalized form (81.6%) of the disease. At the time of the study, all MG patients were on pyridostigmine (240 mg/day), 23 of them used corticosteroids (prednisone 30 mg/day and 12 required immunosuppressive therapy (8 azathioprine, 150 mg/day and 4 mycophenolate mofetil, 1000 mg/day).

### 3.1. Baseline Hemodynamic and Autonomic Data

**Figure 1:**
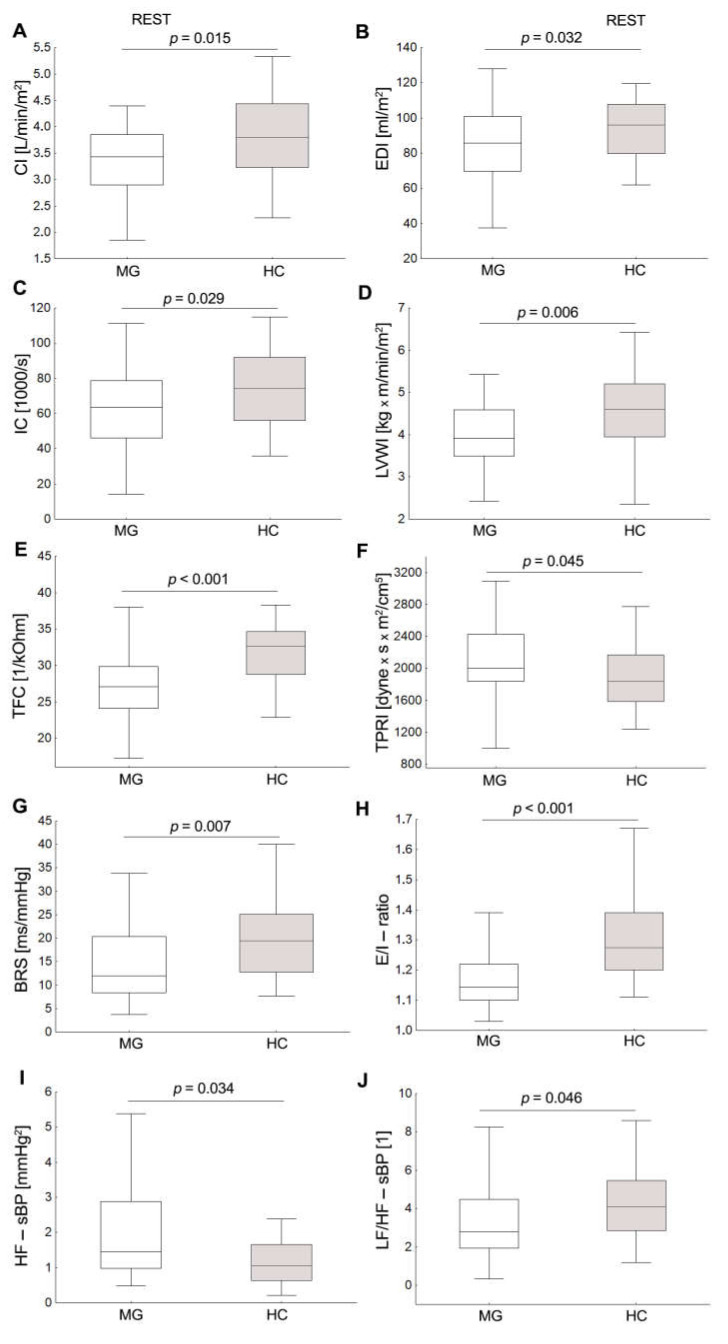
Scatter box plot showing the median, minimum and maximum values of CI, cardiac index (**A**); EDI, end-diastolic index (**B**); IC, index of contractility (**C**); LVWI, left ventricular work index (**D**); TFC, thoracic fluid content (**E**); TPRI, total peripheral resistance index (**F**); BRS, baroreflex sensitivity (**G**); E/I-ratio, expiration/inspiration ratio (**H**); (**I**) HF-sBP, high frequency of systolic blood pressure variability; LF/HF-sBP, the ratio between low and high band for systolic blood pressure variability (**J**) in MG group; respectively for compared to HCs.

All study participants had a normal sinus rhythm. At rest, values for HR, BP, and SV indices were similar in both groups (Table 2). In addition, MG patients as compared to HC were characterized by significantly lower values of cardiac and myocardial contractility parameters, i.e., CI (*p* = 0.015), IC (*p* = 0.029), LVWI (*p* = 0.006), EDI (*p* = 0.032), thoracic fluid content (*p* < 0.001) and significantly higher values of TPRI (0.045). In contrast, no significant differences were observed between the groups in HRV parameters, *p* > 0.05. MG patients had significantly lower BRS and LF/HF-sBP, and higher HF-sBP compared to the HCs (Figure 1).

### 3.2. Cardiovascular Autonomic Function Test

**Figure 2:**
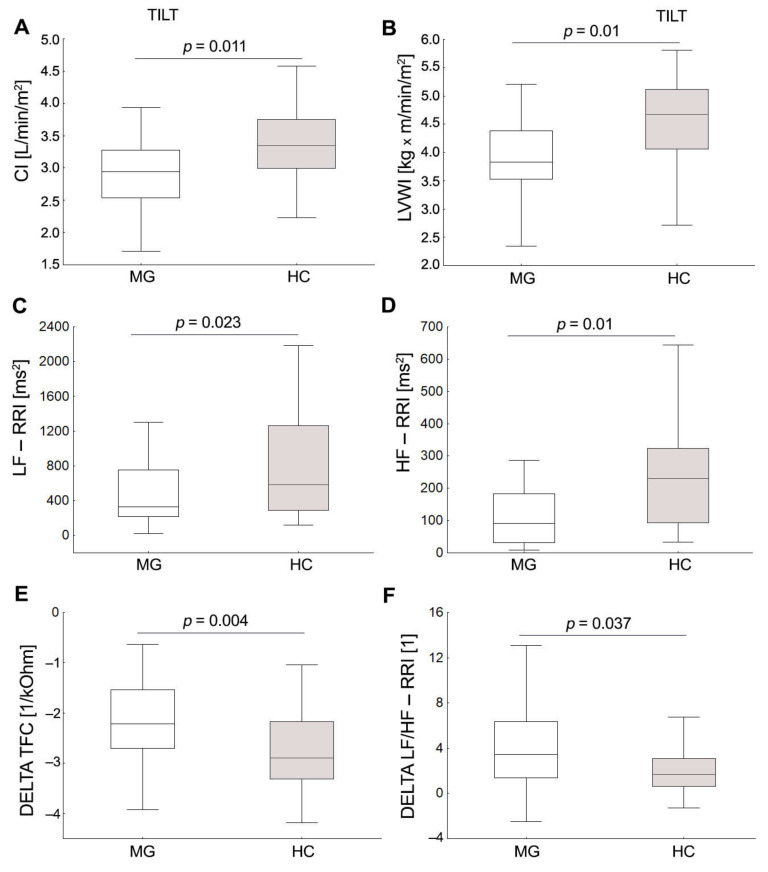
Scatter box plot showing the median, minimum and maximum values of CI, cardiac index (**A**); LVWI, left ventricular work index (**B**); LF-RRI, low frequency of heart rate variability (**C**); HF-RRI, high frequency of heart rate variability (**D**); TFC, thoracic fluid content (**E**); LF/HF-RRI, ratio between low and high band for heart rate variability (**F**) in MG group; respectively for compared to HCs.

None of the subjects had symptoms of orthostatic intolerance in the form of postural tachycardia syndrome and orthostatic hypotension while testing. The cardiac HR and BP response during orthostatic stress was similar in both groups, *p* > 0.05. At 70° tilt, MG patients were characterized by significantly lower values of cardiac and myocardial contractility parameters, i.e., IC (*p* = 0.044), CI, LVWI, as well as lower TFC, HF-RRI, and LF-RRI (Figure 2). Despite similar increases in HR values, the MG group showed greater post-tilt changes in LF/HF-RRI and lowered thoracic fluid content compared to the HCs. In contrast, no significant differences were observed between the groups for post-tilt cardiac and HRV, sBPV parameters, *p* > 0.05. In response to the deep breathing test (DBT), MG patients had a significantly lower E/I-ratio compared to HCs (*p* < 0.001). An abnormal HR response to deep breathing (E/I ratio) was found in 12 (31.6%) of patients and only 1 (3.3%) of the HCs (Figure 1).

### 3.3. Relationship between Clinical and Demographic Features of MG and Cardiac Autonomic, Hemodynamic Parameters

In MG patients, the disease severity as determined by the MGFA classification was positively correlated with disease duration (R = 0.42, *p* = 0.009), HRV parameters, both at rest (LF/HF; R = 0.36 *p* = 0.026), during HUTT (LFnu-RRI, R = 0.33, *p* = 0.042; LF/HF, R = 0.34, *p* = 0.028, LF/HF-RRI; R = 0.35, *p* = 0.036) as well as in response to HUTT (delta LF-RRI, delta HF-RRI, delta PSD-RRI, delta LF/HF-RRI), respectively (Supplementary file, Table S1).

MGFA negatively correlated with thoracic fluid content, both at rest (R = −0.34, *p* = 0.036) as well as during HUTT (R = −0.34, *p* = 0.036). Lower HRV parameters were significantly associated with higher disease severity (LF-RRI, R = −0.42 *p* = 0.009; HF-RRI, R = −0.50 *p* = 0.002, PSD-RRI, R = −0.40, *p* = 0.013). Overall, there was no association between disease duration and autonomic hemodynamic measures, except for delta index of contractility (R = −0.34, *p* = 0.039).

There was a significant negative association between AChR-ab-positive MG patients and LVWI (R = −0.34, *p* = 0.037). There was no significant correlation between AChR-Ab status and the autonomic parameters, *p* > 0.05.

In the multivariable regression model, the disease severity as determined by the MGFA, adjusted for age, and sex was identified as an independent predictor of higher delta LF/HF-RRI (b = 0.34; *p* = 0.043) as well as E/I ratio (b = −0.303; *p* = 0.01) explaining 18% and 15% of the variance, respectively (see Table 3). MGFA (b = −0.44; *p* = 0.005), and age (b = −0.35; *p* = 0.02) were found to predict lower BRS explaining 33% of the variance (see Table 3). Hemodynamic and LV myocardial function parameters (CI, IC, LVWI) were predominantly predicted by gender and age, both in rest and during HUTT. Significant predictors for cardiac, HRV, and SBPV parameters for MG group are presented in Table 3.

## 4. Discussion

In this study, we aimed to compare cardiac and autonomic function in MG patients with mild and moderate severities compared to age-matched healthy subjects, and to explore its relationship with disease status. Our findings showed that MG patients, as compared to controls, were characterized by diminished cardiovagal tone, increased sympathovagal balance of HRV after HUTT, and lower values of BRS at rest. These alterations of cardiac autonomic responses were related to the severity of the disease but not to its duration. Moreover, at rest, MG patients showed significantly altered SBPV and lower LV myocardial function pronounced at rest and during HUTT.

Consistent with previous studies, MG patients had lower HR response to DBT as measured by E/I ratio, reflecting decreased vagal modulation of the sinus node [10,14,26]. Our SBPV findings are partly consistent with previously reported data [20]. No differences between MG patients and controls were observed for SBPV in the LF power either at rest or during HUTT. However, given that the resting HF SBPV increased and the sympathovagal ratio of SBPV decreased, it is reasonable to assume that the increase in HF power may reflect an altered autonomic modulation of systolic blood pressure in patients with MG. In fact, the relevance of HF frequency oscillations is not well understood. The low-frequency oscillations of SBPV reflect the sympathetic activity of the α-adrenergic receptors of the vasculature, whereas high-frequency oscillations probably reflect the mechanical effect of breathing on systolic blood pressure. Other studies suggest the involvement of ANS, particularly the β-adrenergic system, in the HF SBPV component [37]. Thus, our findings of a higher HF band of SBPV, could be associated with an altered respiratory activity on systolic blood pressure variability in MG subjects, however, these results should be interpreted with caution given the heterogeneity and the small number of MG patients in this analysis. At rest, the HRV frequency-domain parameters were similar in both groups. It has been shown that use of acetylcholine esterase inhibitors is associated with increasing the concentration of acetylcholine both in the synaptic cleft in the neuromuscular junction and in synapses of CNS and autonomic ganglia. Experimental evidence indicates that pyridostygmine improves the autonomic profile, reduces HR at rest, and enhances short-term HRV [38,39,40]. A previous study in spontaneously hypertensive rats showed that cholinergic stimulation with pyridostigmine bromide for 2 weeks reduced BP, HR as well as increased vagal participation in autonomic balance and decreased systolic BPV [38]. The mechanisms involved in SBPV findings remain unclear and require further investigation.

In response to head-up tilt, both MG and control groups demonstrated similar HR and sBP responses, whereas the increase in the sympathovagal ratio of HRV was higher in patient groups, indicating an autonomic modulation shift towards sympathetic hyperactivity. In addition, orthostatic dysregulation was also reflected in greater reduction of total power density of HRV as well as LF and HF power decrease when expressed in absolute units compared with HCs. This represents an overall decrease o of cardiovagal activity, as well as combined sympathetic and parasympathetic cardiac activity [41].

Sympathetic hyperresponsiveness was found in different groups of patients with MG [14], with and without thymoma [19], and those in myasthenic crises [20]. In a study of 64 MG patients, Shukla et al. showed a significantly higher HR and BP response during 3 min of 70° head-up tilt test and hand grip maneuvers suggesting sympathetic dysfunction with abnormal hyperactivity to stress. Furthermore, they found no significant difference in parasympathetic function expressed as Valsalva ratio and R–R interval variability [19].

These findings were similar to those reported by Kocabas et al. in a study of 30 MG patients who found higher sBP and dBP, both in rest and during the tilt test in MG compared to HCs. They concluded that sympathovagal balance has been disturbed in favor of sympathetic tone, and parasympathetic insufficiency has become more prominent both at rest and during the tilt phase, however, autonomic function using Ewing’s battery test did not show a significant difference between the patient and the control group [14]. Another study in 21 MG patients with thymoma using 24-h Holter monitoring, HRV analysis, and Ewing’s battery tests have reported a tendency toward LFnu-RRI and LF/HF-RRI increase and HFnu-RRI decrease at rest, which is in line with our study. As a result, the authors hypothesized that Ewing’s battery tests showed mainly parasympathetic dysfunction, however, the sympathetic arm of ANS seemed to be slightly affected [14]. These reports confirm our previous findings, which indicate cardiac parasympathetic impairment as well as sympathovagal imbalance in favor of sympathetic tone in MG patients and relatively preserved sympathetic function in response to orthostatic stress [16].

Several studies suggest the presence of complex interactions between disease severity, ANS dysregulation, and chronic inflammation [42]. The imbalance in the ANS may favor inflammation when the SNS is dominant [43,44]. In our study, greater disease severity, as determined by MGFA corrected for age and sex, was an independent predictor of diminished vagal tone (E/I ratio) and increased sympathetic response to tilt (delta LF/HF-RRI) as measured with HRV. This may reflect that patients in the advanced disease stage paralleled the lower parasympathetic activity as well as sympathetic overactivity related to chronic inflammation.

In keeping with our findings, sympathetic hyperactivity may be an important factor in hyperinflammation with cytokine storm [12].

Consistent with previous studies [10,14,15], MG patients had lower values of BRS at rest which reflects reduced vagal outflow and increased sympathetic outflow to the heart.

Another possible explanation of sympathetic overactivity observed in MG patients may be associated with diminished central autonomic regulation in the BRS. Studies indicate that decreased baroreflex sensitivity may contribute to cardiac conduction abnormalities as well as increased risk for cardiovascular diseases through an impairment of the cholinergic anti-inflammatory pathway [42]. In our study, we showed that older age and greater disease severity turned out to be predictors of lower BRS. Thus, it could be speculated that cardiac sympathetic hyperactivity has greater clinical relevance in the more severely affected patients (i.e., MGFA class IVB or class V) and those with advancing age and late-onset of MG disease (>50 years) [6]. Aging is a main risk factor for neurodegeneration. Chronic activation of microglial and astrocytes may play a role in neuroinflammation, leading to CNS neurodegeneration, including the hypothalamus, which represents the control centers for sympathetic and parasympathetic outflows [45,46,47]. In our study, a higher age and female sex were significant predictors of resting TPRI increase and CI reduction during tilt, whereas older age was an independent predictor of decreased LV myocardial function and PSD-RRI both at rest and during tilt.

As previously described, MG is associated with a higher prevalence of cardiac manifestations, ranging from asymptomatic electrocardiographic changes to myocarditis, heart failure, and cardiac arrhythmia. A literature review conducted by Shivamurthy et al. confirmed that older age, severe myasthenia, and myocarditis appeared to be associated with anti-striational antibodies (e.g., against titin, ryanodine receptor, or voltage-gated potassium channel) [6]. Our findings support the presence of subclinical cardiac involvement in stable MG patients treated with pyridostigmine which was not related to the disease severity [20].

Using ICG, we have demonstrated that MG patients’ demographics were associated with impaired cardiac function, including subtle reductions in LV myocardial function and decreases in thoracic fluid content pronounced at rest and during HUTT. In addition, the presence of AChR-abs was associated with lower LVWI, which may suggest an underlying autoimmune mechanism of reduced cardiac inotropy parameters in MG.

Changes in left ventricular work index paralleled changes in cardiac index, thus, reduction in cardiac output or blood pressure or a combination of both could be compensated by sympathetic α-adrenergic vasoconstriction expressed as an increase in TPR.

Similar to our study, Perez et al. found a similar pattern of hemodynamic changes with a decrease in cardiac index accompanied by an increase in TPRI in half of patients with MG and thymoma. They also postulated increased incidence of atrial fibrillation, ventricular and supra ventricular extra systoles and prolonged corrected QT interval [14].

Using variables derived from ECG and echocardiography, Kato et al., found that a small number of patients without ECG abnormality demonstrated cardiac damage, which comprised only diastolic impairment (E/e ratio) and did not include systolic impairment [25]. In the current study, a low TFC value could be an indirect measure of reduced cardiac preload and/or hypovolemia as a cause of fatigue and dyspnoea. Low preload as a cause of dyspnoea was previously reported in patients with autonomic diseases and postural orthostatic [46,48,49].

Our study has several clinical implications. Firstly, CAD symptoms should be considered when evaluating MG patients, especially those with an advanced disease stage. Secondly, we have shown that diminished cardiovagal tone and sympathetic hyperresponsiveness are present in MG patients despite the enhancement of vagal tone by the use of acetylcholine esterase inhibitors. Thus, the parasympathetic deficit is underestimated in MG patients and is more extensive than the disturbances of cholinergic transmission at the neuromuscular junction. Furthermore, as the severity of the disease increases, the lower parasympathetic activity is observed, which confirms the strong association between CAD and the immune response. It is noteworthy that by contributing to the reduction of MG severity, we also reduce their risk of cardiovascular events. On the other hand, clinicians should be aware that MG patients with severe disease are more at risk of cardiovascular complications than those with minimal manifestation. Moreover, it can be assumed that autonomic imbalance and LV myocardial function abnormalities may contribute to the lower exercise capacity of MG patients, which is a common feature of this condition.

A few limitations of this study should be addressed. First, we did not include patients who were in stage IIIb-V at MGFA classification, which significantly limited the analysis of the overall disease impact on cardiovascular risk. Second, our study did not assess as a subgroup of MG, MUSK-Ab-positive, since they are present in a small proportion of patients. Third, our study is cross-sectional and no longitudinal data are available. Furthermore, we did not take into account the impact of treatment with pyridostygmine, steroids, and azathioprine on cardiac and autonomic parameters. Our study includes pre-diabetic conditions, however, prediabetes is found to be also associated with dysfunction of cardiac autonomic activity and includes decreased HRV, and reduced parasympathetic modulation of the heart [50]. We evaluated only clinically stable patients, so it was not possible to evaluate the impact of a possible exacerbation of the disease on CAD parameters. Finally, the sample size was relatively small, but many previous studies have involved fewer patients than in our group.

Finally, future research should consider assessing both linear and non-linear measures of HRV and BPV, which can be a promising field for exploring autonomic cardiac loops of circulation in MG patients. Several studies have shown that such methods were useful tools for clinical practice, in particular, in patients with cardiovascular events (hypertension, myocardial infarction) [51,52,53,54].

## 5. Conclusions

In conclusion, cardiac autonomic dysfunction is associated with predominant parasympathetic impairment in patients with myasthenia gravis. Alterations of cardiac autonomic responses were related to the severity of the disease but not to its duration. Moreover, MG patients were characterized by altered SBPV and lower subclinical LV myocardial function. Clinicians should consider the evaluation of CAD in MG patients with, or at risk for, cardiovascular disease, especially in advanced stages of the disease. Non-invasive monitoring of beat-to-beat BPV and HRV may be helpful in providing insight into the early deteriorating mechanisms of cardiovascular autonomic control.

## Figures and Tables

**Table 1:** Clinical and demographic baseline characteristics of patients.

	MG Patients	HC	*p*-Value
Number of subjects	38	30	
Sex (male/female),	5/33	7/23	0.274
Age, years, median	40.5 (19–69)	35.5 (26–59)	0.054
Age at first manifestation, median, (years)	32.0 (12–68)		
Early-onset MG (<50 years)	35 (92.1%)		
Disease duration (years), median (range)	5.5 (0.5–24)		
First symptom, *n* (%)			
Seropositivity to AchR antibodies, *n* (%)	23 (60.5%)		
Seropositivity to MuSK antibodies	0 (0%)		
Type of MG, *n* (%)			
Ocular	7 (18.4%)		
Generalized	31 (81.6%)		
Thymectomy, *n* (%)	14 (36.8%)		
Severity of disease at the moment of testing (MGFA, %)			
Class 0	0		
Class I (ocular)	8 (21.1%)		
Class IIa	19 (50.0%)		
Class IIIa	11 (28.9%)		
Histology changes, *n* (%)			
Thymic pathology	22 (57.9%)		
Thymoma	1 (2.7%)		
Type of treatment			
Use of an anticholinesterase	38 (100%)		
Use of corticosteroids	23 (60.5%)		
Immunosupressive	13 (34.2%)		

Myasthenia gravis (MG), healthy controls (HC), Acetylcholine receptor (AchR), muscle-specific kinase (MuSK), Myasthenia Gravis Foundation of America (MGFA).

**Table 2:** Median (lower quartile–upper quartile) of hemodynamic and autonomic parameters for MG patients and healthy controls.

Group	MG	HC	MG	HC	MG	HC
	Rest		70° Tilt		Change after 70° Tilt (Delta)	
Hemodynamic measures						
HR [1/min]	62.5 (59.8,69.7)	68.8 (61.5,71.1)	75.6 (69.9,83.6)	82.2 (75.8,86.9)	10.4 (6.7,16.9)	14.3 (10.4,18.1)
sBP [mmHg]	113.0 (106.7,120.2)	110.0 (103.0,121.2)	120.2 (116.7,126.6)	122.8 (117.5,132.9)	6.0 (1.6,19.4)	14.3 (6.7,20.0)
dBP [mmHg]	72.6 (67.5,75.9)	73.7 (66.9,78.0)	85.7 (78.5,88.9)	89.9 (83.3,92.9)	12.4 (7.8–17.0)	15.8 (8.5,21.2)
mBP[mmHg]	89.7 (85.0,93.9)	90.9 (81.3,97.7)	99.1 (95.5,104.8)	103.8 (97.2,110.3)	10.6 (5.6,16.0)	14.4 (8.8,21.1)
SI [ml/m^2^]	55.3 (44.1,61.1)	59.2 (51.1,66.0)	39.0 (34.5,42.5)	41.2 (36.7,45.6)	−16.2 (−20.3,−9.1)	−17.7 (−22.9,−13.6)
CI [l/min/m^2^]	3.4 (2.9,3.9)	3.8 (3.2,4.4) *	2.9 (2.5,3.3)	3.3 (3.0,3.8) *	−0.5 (−0.8,−0.2)	−0.6 (−0.8,−0.5)
TPRI [dyn × s m^2^/cm^5^]	20004.7 (1838.9–2428.6)	1838.0(1582.6,2167.8) *	2627.9 (2365.2,3250.5)	2458.6 (2136.3,2921.7)	613.0 (249.6,928.3)	670.0 (392.8,1004.8)
LVWI [kg m/min/m^2^]	3.9 (3.5,4.6)	4.6(4.0,5.2) *	3.8 (3.5,4.4)	4.7 (4.1,5.1) *	−0.2 (−0.5,0.2)	0.0 (−0.5,0.4)
IC [1000/s]	63.7 (46.2,78.6)	74.3 (56.2,92.0) *	46.8 (35.9,53.9)	52.9 (44.0,62.2)	−18.7 (−28.0,−6.9)	−23.9 (−31.1,−17.4)
EDI [ml/m^2^]	85.8 (69.8,100.7)	96.1 (79.7,107.6)	69.8 (61.3,76.5)	75.6 (67.3,84.2)	−18.6 (−25.7,−8.7)	−21.1 (−28.1,−12.9)
TFC [1/kOhm]	27.1 (24.1,29.9)	32.6 (28.7,34.6) *	24.6 (22.6,27.5)	29.4 (26.6,32.1)	−2.2 (−2.7,−1.5)	−2.9 (−3.3,−2.2) *
Heart rate variability (HRV)						
LFnu-RRI [ms^2^]	61.1 (46.8,73.8)	58.5 (49.2,65.4)	82.9 (67.4,89.5)	76.9 (65.0,81.7)	17.6 (11.1,29.4)	12.9 (6.3,22.5)
HFnu-RRI [ms^2^]	38.9 (26.2,53.2)	41.5 (34.6,50.8)	17.1 (10.5,32.6)	23.1 (18.3,35.0)	−17.6 (−29.4,−11.1)	−12.9 (−22.5,−6.3)
LF-RRI [ms^2^]	570.3 (310.3,1240.6)	614.8 (392.4,1117.0)	331.6 (217.9,755.1)	585.7 (291.1,1262.7) *	−85.1 (−543.7,30.0)	−78.1 (−220.6,232.2)
HF-RRI [ms^2^]	289.3 (102.6,1012.5)	364.0 (198.9,882.1)	90.9 (32.5,183.7)	230.8 (92.9,324.4) *	−172.4 (−797.3,−67.1)	−161.5 (−640.6,−49.4)
PSD-RRI [ms^2^]	1246.8 (709.4,2843.7)	1420.4 (855.8,2458.8)	762.9 (403.7,1362.7)	1080.1 (615.1,2096.2)	−447.7 (−1754.4,−145.9)	−443.5 (−1088.6,−57.8)
LF/HF-RRI [1]	1.7 (0.9,3.0)	1.5 (1.0,2.0)	5.2 (2.2,9.4)	3.4 (1.9,5.0)	3.4 (1.4,6.3)	1.7 (0.6,3.1) *
LF/HF [1]	1.1 (0.7,1.8)	1.1 (0.9,1.5)	3.0 (1.3,5.5)	2.5 (1.3,3.6)	1.8 (0.4,4.2)	1.2 (0.4,2.5)
Systolic Blood pressure variability (SBPV)						
LFnu-sBP [%]	41.2 (31.9,48.1)	40.2 (35.7,45.8)	51.1 (41.3,62.2)	46.5 (38.1,54.7)	10.1 (4.8,17.1)	11.0 (1.0,17.6)
HFnu-sBP [%]	12.5 (9.0,19.6)	10.7 (6.9,15.4)	15.0 (10.7,20.4)	13.6 (8.3,18.4)	1.6 (−0.8,3.7)	2.1 (−0.0,3.2)
LF-sBP [mmHg^2^]	4.5 (3.4,8.0)	3.6 (2.9,7.2)	4.2 (2.7,6.6)	3.4 (2.1,6.3)	−0.3 (−1.6,0.4)	−0.4 (−1.6,0.1)
HF-sBP [mmHg^2^]	1.4 (1.0,2.9)	1.1 (0.6,1.7)*	1.1 (0.9,2.3)	1.0 (0.5,1.6)	−0.2 (−0.5,0.0)	−0.1 (−0.5,0.0)
PSD-sBP [mmHg^2^]	12.1 (7.8,17.3)	10.4 (7.7,15.7)	8.4 (6.3,11.9)	7.8 (5.7,12.4)	−2.6 (−6.7,−1.1)	−2.6 (−5.2,−1.2)
LF/HF-sBP [1]	2.8 (1.9,4.5)	4.1 (2.9,5.5) *	3.3 (2.1,5.0)	4.0 (2.4,5.2)	0.0 (−0.4,0.7)	0.1 (−0.7,0.6)
BRS [ms/mmHg]	12.0 (8.4,20.3)	19.4 (12.8,25.2) *				

Myasthenya gravis (MG), healthy controls (HC), heart rate (HR), systolic blood pressure (sBP), diastolic blood pressure (dBP), mean blood pressure (mBP), stroke index (SI), cardiac index (CI), total peripheral index (TPRI), left ventricular work index (LVWI), thoracic fluid content (TFC), end-diastoic index (EDI), index of contractility (IC), low frequency R-R interval (LF-RRI), high-frequency R-R interval (HF-RRI), power spectral density R-R interval (PSD-RRI), ratio between low and high band for heart rate variability (LF/HF-RRI) ratio between low and high band for heart rate and blood pressure variability (LF/HF), power spectral density of systolic blood pressure variability (PSD-sBP), low frequency of systolic blood pressure variability (LF-sBP), high frequency of systolic blood pressure variability (HF-sBP), power spectral density of diastolic blood pressure variability (PSD-sBP), the ratio between low and high band for systolic blood pressure variability (LF/HF-sBP), baroreflex sensitivity (BRS); nu, normalised values; statistically significant differences are indicated with * *p* < 0.05.

**Table 3:** Multivariate analysis for prediction of frequency domain and cardiac and autonomic variables by clinical features.

Dependent Variables	Model Variables	BETA (β)	SE	*p*-Value	R^2^
E/I-ratio	MGFA	−0.36	0.16	0.034	0.15
HF-sBP	Sex	−0.35	0.17	0.042	0.14
BRS	MGFA	−0.44	0.15	0.005	0.33
	Age	−0.35	0.14	0.020	
CI	Sex	0.33	0.16	0.047	0.17
TPRI	Age	0.47	0.14	0.002	0.35
	Sex	−0.40	0.14	0.002	
EDI	Age	−0.47	0.15	0.003	0.26
IC	Age	−0.41	0.15	0.011	0.21
Tilt CI	Age	−0.68	0.12	<0.001	0.52
	Sex	0.30	0.12	0.024	
Tilt LVWI	Age	−0.61	0.13	<0.001	0.45
Tilt LF-RRI	Age	−0.50	0.15	0.002	0.25
Tilt PSD-RRI	Age	−0.37	0.16	0.028	0.16
Tilt TFC	Sex	−0.32	0.15	0.043	0.30
DELTA LF/HF-RRI	MGFA	0.34	0.16	0.047	0.18

Expiration/inspiration ratio (E/I–ratio), high frequency of systolic blood pressure variability (HF-sBP), baroreflex sensitivity (BRS), cardiac index (CI), total peripheral resistance index (TPRI), end-diastolic index (EDI), index of contractility (IC), left ventricular work index (LVWI), low frequency of heart rate variability (LF-RRI), power spectral density R-R interval (PSD-RRI), thoracic fluid content (TFC), ratio between low and high band for heart rate variability (LF/HF-RRI), standardized beta coefficient (β ), standard error (SE), R, squared (R^2^).

## Data Availability

All the data are presented within the manuscript.

## Supplementary Materials

The following supporting information can be downloaded at: https://www.mdpi.com/article/10.3390/jcm11133697/s1, Table S1: Association of cardiovascular and autonomic measures with MG and clinical outcomes.
