# Supplementary material for: Association of Cardiac Autonomic Responses with Clinical Outcomes of Myasthenia Gravis: Short-Term Analysis of the Heart-Rate and Blood Pressure Variability
Source: J Clin Med. 2022 Jun 27;11(13):3697. doi: 10.3390/jcm11133697 (PMC9267657; doi:10.3390/jcm11133697)
Supplement: Supplementary file 1 [file jcm-11-03697-s001.zip › jcm-1761852-supplementary.pdf]

Parameter	Disease duration	MGFA	AChR-Ab status
HR	0.13	0.25	-0.01
sBP	-0.04	-0.01	-0.06
dBP	-0.08	0.07	-0.21
mBP	-0.07	0.07	-0.22
SI	0.13	-0.06	-0.14
CI	0.28	0.14	-0.24
TPRI	-0.23	-0.05	0.08
LVWI	0.17	0.11	-0.34*
TFC	0.19	-0.34*	-0.08
EDI	0.15	-0.10	-0.10
IC	0.25	-0.08	-0.14
LFnu-RRI	0.20	0.31	0.10
HFnu-RRI	-0.20	-0.31	-0.10
LF-RRI	-0.13	-0.42**	-0.09
HF-RRI	-0.18	-0.50**	-0.10
PSD-RRI	-0.04	-0.40*	-0.05
LF/HF-RRI	0.22	0.30	0.12
LF/HF	0.21	0.36*	0.03
LFnu-sBP	0.07	0.03	-0.14
HFnu-sBP	-0.10	-0.07	0.05
LF-sBP	0.21	-0.05	0.03
HF-sBP	-0.04	-0.08	0.06
PSD-sBP	0.12	-0.14	0.00
LF/HF-sBP	0.05	0.08	-0.12
BRS	0.18	-0.26	-0.08
**TILT**			
HR	0.01	0.08	0.10
sBP	-0.01	0.12	-0.14
dBP	-0.00	0.09	-0.15
mBP	-0.02	0.13	-0.18
SI	0.09	0.05	0.00
CI	0.12	0.04	-0.03
TPRI	-0.09	-0.01	0.02
LVWI	0.07	0.13	-0.10
TFC	0.17	-0.36*	-0.10
EDI	0.06	-0.00	0.00
IC	0.13	0.01	-0.02
LFnu-RRI	0.15	0.33*	0.14
HFnu-RRI	-0.15	-0.33*	-0.14
LF-RRI	-0.05	-0.09	-0.03
HF-RRI	-0.15	-0.34*	-0.13
PSD-RRI	-0.07	-0.16	-0.05
LF/HF-RRI	0.17	0.36*	0.14
LF/HF	0.19	0.34*	0.12
LFnu-sBP	0.13	0.07	-0.08
HFnu-sBP	-0.11	-0.15	0.16
LF-sBP	0.19	0.03	0.04
HF-sBP	-0.04	-0.16	0.11
PSD-sBP	0.14	0.04	0.03
LF/HF-sBP	0.12	0.15	-0.13

DELTA (change tilt-supine)			
HR	-0.12	-0.22	0.13
sBP	-0.03	0.10	-0.04
dBP	0.00	0.03	-0.02
mBP	0.02	0.05	0.04
SI	-0.19	0.05	0.21
CI	-0.25	-0.09	0.23
TPRI	0.03	0.01	-0.20
LVWI	-0.14	-0.10	0.21
TFC	-0.07	0.24	0.07
EDI	-0.29	0.04	0.13
IC	-0.34*	0.07	0.17
LFnu-RRI	-0.02	-0.04	-0.02
HFnu-RRI	0.02	0.04	0.02
LF-RRI	0.11	0.37*	0.11
HF-RRI	0.13	0.35*	0.10
PSD-RRI	0.00	0.39*	0.00
LF/HF-RRI	0.11	0.36*	0.15
LF/HF	0.08	0.27	0.14
LFnu-sBP	0.13	0.09	0.00
HFnu-sBP	-0.05	-0.16	0.15
LF-sBP	0.01	0.20	-0.08
HF-sBP	0.06	0.02	0.02
PSD-sBP	-0.06	0.22	-0.06
LF/HF-sBP	-0.04	0.01	-0.22

Supplementary file

Table S1. Association of cardiovascular and autonomic measures with MG and clinical outcomes

Myasthenia Gravis Foundation of America (MGFA), AChR autoantibody (AChR-Ab), heart rate (HR), systolic blood pressure (sBP), diastolic blood pressure (dBP), mean blood pressure (mBP), stroke index (SI), cardiac index (CI), total peripheral index (TPRI), left ventricular work index (LVWI), thoracic fluid content (TFC), end-diastoic index (EDI), index of contractility (IC), low frequency R-R interval (LF-RRI), high-frequency R-R interval (HF-RRI), power spectral density R-R interval (PSD-RRI), ratio between low and high band for heart rate variability (LF/HF-RRI) ratio between low and high band for heart rate and blood pressure variability (LF/HF), power spectral density of systolic blood pressure variability (PSD-sBP), low frequency of systolic blood pressure variability (LF-sBP), high frequency of systolic blood pressure variability (HF-sBP), power spectral density of diastolic blood pressure variability (PSD-sBP), the ratio between low and high band for systolic blood pressure variability (LF/HF-sBP), baroreflex sensitivity (BRS); nu, normalised values; statistically significant differences are indicated with *p<0.05, **p<0.01.
